# Genetically Engineered Pigs as Efficient Salivary Gland Bioreactors for Production of Therapeutically Valuable Human Nerve Growth Factor

**DOI:** 10.3390/cells11152378

**Published:** 2022-08-02

**Authors:** Fang Zeng, Sha Liao, Zhe Kuang, Qingchun Zhu, Hengxi Wei, Junsong Shi, Enqin Zheng, Zheng Xu, Sixiu Huang, Linjun Hong, Ting Gu, Jie Yang, Huaqiang Yang, Gengyuan Cai, Stefan Moisyadi, Johann Urschitz, Zicong Li, Zhenfang Wu

**Affiliations:** 1 National Engineering Research Center for Breeding Swine Industry, South China Agricultural University, Guangzhou 510642, China; fangzeng@scau.edu.cn (F.Z.); liaosha@stu.scau.edu.cn (S.L.); kuangzhe@stu.scau.edu.cn (Z.K.); qingchun.z@stu.scau.edu.cn (Q.Z.); weihengxi@scau.edu.cn (H.W.); eqzheng@scau.edu.cn (E.Z.); stonezen@scau.edu.cn (Z.X.); sxhuang815@scau.edu.cn (S.H.); linjun.hong@scau.edu.cn (L.H.); tinggu@scau.edu.cn (T.G.); jieyang2012@hotmail.com (J.Y.); yangh@scau.edu.cn (H.Y.); cgy0415@scau.edu.cn (G.C.); 2 Department of Animal Genetics, Breeding and Reproduction, College of Animal Science, South China Agricultural University, Guangzhou 510642, China; 3 Department of Aquaculture, College of Marine Science, South China Agricultural University, Guangzhou 510642, China; 4 Guangdong Provincial Key Laboratory of Agro-Aniamal Genomics and Molecular Breeding, South China Agricultural University, Guangzhou 510642, China; 5 Guangdong Wens Pig Breeding Technology Co., Ltd., Yunfu 527499, China; junsongstone@stu.scau.edu.cn; 6 Institute for Biogenesis Research, John A. Burns School of Medicine, University of Hawaii at Manoa, Honolulu, HI 96822, USA; moisyadi@hawaii.edu (S.M.); johann@hawaii.edu (J.U.); 7 State Key Laboratory for Conservation and Utilization of Subtropical Agro-Bioresources, South China Agricultural University, Guangzhou 510642, China; 8 Guangdong Provincial Laboratory of Lingnan Modern Agricultural Science and Technology, Guangzhou 510642, China

**Keywords:** bioreactors, expression systems, transgenic pigs, salivary glands, nerve growth factor

## Abstract

Farm animal salivary glands hold great potential as efficient bioreactors for production of human therapeutic proteins. Nerve growth factor (NGF) is naturally expressed in animal salivary glands and has been approved for human clinical treatment. This study aims to employ transgenic (TG) pig salivary gland as bioreactors for efficient synthesis of human NGF (hNGF). hNGF-TG pigs were generated by cloning in combination with piggyBac transposon-mediated gene transfer. These hNGF-TG pigs specifically expressed hNGF protein in their salivary glands and secreted it at high levels into saliva. Surgical and nonsurgical approaches were developed to efficiently collect saliva from hNGF-TG pigs. hNGF protein was successfully purified from collected saliva and was verified to be biologically active. In an additional step, the double-transgenic pigs, where the endogenous porcine NGF (pNGF) gene was replaced by another copy of *hNGF* transgene, were created by cloning combined with CRISPR/Cas9-mediated homologous recombination. These double-transgenic pigs expressed hNGF but not pNGF, thus avoiding possible “contamination” of hNGF with pNGF protein during purification. In conclusion, TG pig salivary glands can be used as robust bioreactors for a large-scale synthesis of functional hNGF or other valuable proteins. This new animal pharming method will benefit both human health and biomedicine.

## 1. Introduction

Transgenic (TG) farm animals are efficient but low-cost “drug factories” that can be employed to synthesize therapeutically important human proteins with high qualities [1,2,3,4]. To date, three pharmaceutical proteins produced from the milk of TG goats, the milk of TG rabbits, and the egg white of TG chickens, respectively, have been approved in the USA, Europe, and some other countries for human clinical use [2,5], which have brought significant positive impacts on human health and biomedicine. While farm animal mammary gland and oviduct bioreactors hold promise for therapeutical protein production, they have several disadvantages: (1) only female but not male TG farm animals can synthesize foreign proteins from the milk or eggs; (2) female TG farm animals are unable to produce heterologous proteins in their milk or eggs when they are in the non-lactating or non-laying period; and (3) farm animal mammary glands or oviducts may be non-tolerant or sensitive to some expressed foreign proteins, which may cause serious side effects on host TG farm animals. Hence, there is a need to develop new farm animal bioreactor systems for the efficient production of therapeutic proteins.

Livestock salivary glands hold potential as efficient bioreactors, as both male and female farm animals are naturally able to continuously synthesize and secrete diverse biologically active proteins into saliva during their entire life span [6,7,8]. Additionally, many adult farm animals secrete a large volume of saliva every day. For example, pigs, goats, sheep, and cows can produce an average of 15, 6–16, 6–16, and 60–190 L of saliva per day, respectively [9,10,11]. More importantly, saliva can be collected in a large quantity from various livestock such as cattle, sheep, and goats, either by surgical or nonsurgical methods [11,12,13,14], facilitating the large-scale purification of target proteins from saliva. These advantages collectively suggest that livestock salivary glands may serve as robust bioreactors for therapeutic protein production.

Nerve growth factor (NGF) is a therapeutically important protein that was first identified by Cohen and Levi-Montalcini, who were awarded the 1986 Nobel Prize for Physiology or Medicine for the discovery of NGF [15,16]. NGF plays a vital role in the growth, differentiation, and regeneration of nerve cells. It is not only effective for the treatment of various neuronal ailments such as glaucoma and Alzheimer’s disease but also has promising therapeutic potential for some non-neuronal disorders such as vascular and immune diseases [17,18,19,20,21]. Human NGF (hNGF) synthesized by *E. coli* recently has been approved in the USA and Europe for clinical treatment of a corneal nerve impairment disease called neurotrophic keratitis in humans [22]. However, bacteria-expressed hNGF may exhibit a lower activity than mammalian cell-produced hNGF because bacteria usually are unable to provide proper post-translational modifications for human proteins [3,23,24]. Mouse NGF (mNGF) isolated from mouse salivary glands has a high homology to hNGF and was approved in China for treating several nerve damage diseases in humans [25]. However, mNGF also showed a lower stability and weaker bioactivity than hNGF [26]. Therefore, hNGF protein produced and purified from mammalian expression platforms should be an attractive pharmaceutical for human clinical use. Livestock salivary glands are well-suited for the synthesis of functional hNGF, since biologically active NGF is naturally expressed in the salivary glands of mammals [27,28,29,30], suggesting that salivary glands can provide correct assembly of NGF and are tolerant to NGF.

In this study, we used genetically modified pigs as powerful salivary gland bioreactors to produce functional hNGF protein from the saliva, thus providing a new and efficient strategy for production of human therapeutic proteins.

## 2. Materials and Methods

### 2.1. Ethics Statement

The animal experimental protocol (no. SYXK 2014-0136) of this study was approved by the Institutional Animal Care and Use Committee of the South China Agricultural University. All efforts were made to minimize animal suffering.

### 2.2. Plasmid Construction

The pmPSP-hNGF-EGFP plasmid, also named pmPSP-hNGF plasmid, was reported in a previous study [31]. pmPB was a kind gift from the Wellcome Trust Sanger Institute (Cambridgeshire, UK), which was constructed as previously described [32]. Eleven designed pNGF-targeting sgRNAs (for sequences, see Supplementary Table S3) were synthesized and inserted respectively into the Bpi I restriction site of pX330-U6-Chimeric-BB-CBh-hSpCas9 plasmid (Addgene, Catalog no. 42230) to construct 11 corresponding ppNGF-sgRNA-Cas9 plasmids. A 4.3 kb DNA fragment containing the LA, hNGF-bGH-pA, pCMV-Neo-2A-RFP-bGH-pA, and RA was synthesized and inserted between the Xba I and Hind III restriction sites of pUC57 plasmid (GenScript, Catalog no. SD1176) to construct the pLA-hNGF-RFP-RA plasmid.

### 2.3. Genetically Modified Donor Cell Selection

Trypsin-digested pig fibroblasts were co-transfected with the pmPSP-hNGF-EGFP and the pmPB plasmids, or with pLA-hNGF-RFP-RA and ppNGF-sgRNA5-Cas9/ppNGF-sgRNA10-Cas9 plasmids by electroporation. Transfected fibroblasts were diluted with DMEM complete medium containing 10% FBS and seeded on 10 cm dishes with a density of about 200 cells/dish. At 2 to 3 days post-transfection, 1 mg/mL of G418 was added to the cell culture medium for the selection of TG fibroblasts. When well-separated cell colonies expanded to about 1 cm in diameter, EGFP-expressing or RFP-expressing cell colonies were isolated by cloning cylinders and transferred to 24-well plates for further culture. When cells grew to near confluence, they were transferred to 6-well plates and then to 6 cm dishes for further growth.

### 2.4. Somatic Cell Nuclear Transfer

Ovaries of gilts were obtained from a local slaughterhouse and transported to the laboratory in 0.9% saline (*w/v*) supplemented with penicillin-G (100 IU/mL) and streptomycin sulfate (100 mg/L) at 30 °C–35 °C. Follicular fluid with the oocytes was aspirated from the antral follicles (3–6 mm in diameter) using an 18-gauge needle connected to a 10 mL syringe. Cumulus-oocyte complexes (COCs) with at least three layers of compact cumulus cells and a homogenous cytoplasm were selected for in vitro maturation (IVM). Approximately 50–60 selected COCs were transferred to each well of a 4-well Nunc dish with 500 μL IVM medium [33], and cultured at 38.5 °C with 5% CO_2_ in a humidified atmosphere for 44 h. The COCs then were treated with DPBS containing 1 mg/mL hyaluronidase to remove the surrounding cumulus cells. The oocytes with the first polar body in the perivitelline space were considered as matured oocytes. The in vitro oocyte maturation rate in this study was around 67%. The matured oocytes with intact cell membranes and clear perivitelline space were selected for subsequent SCNT.

The matured oocytes and a small number of donor cells were suspended in T2 medium (TCM-199 medium with 2% FBS) containing 7.5 μg/mL cytochalasin B (CB). The oocytes were enucleated by using a 17 μm diameter needle (Lingen Precision Medical Products Co., Ltd., Shanghai, China). The needle was inserted into the oocyte to aspirate the first polar body along with approximately 15% of the adjacent cytoplasm that contained the genomic DNA. Enucleated oocytes were stained with 1 g/mL Hoechst 33,342 and examined under UV light irradiation. Only those completely enucleated oocytes were used for subsequent nuclear transfer. The enucleation rate in this study is about 89%. Donor cells with a round and slightly burr-like shape were aspirated into the injection needle, and one donor cell was injected into the perivitelline space of each enucleated oocyte. The reconstructed embryos were washed three times in PZM-3 medium [34], and then electrically fused using two direct current pulses of 150 V/mm for 50 µs in 0.28 mol/L mannitol supplemented with 0.1 mM MgSO_4_ and 0.01% PVA. The fusion rate in the present usually reached approximately 95%. The fused embryos were cultured in PZM-3 medium for 1 h and then activated by two direct current pulses of 100 V/mm for 20 µs in 0.28 mol/L mannitol supplemented with 0.1 mM MgSO_4_ and 0.05 mM CaCl_2_. Activated embryos were transferred into PZM-3 medium supplemented with 5 mg/mL CB for 4 h and then cultured in fresh PZM-3 medium at 38.5 °C, 5% CO_2_, and saturated humidity.

The two cell-stage embryos were selected at 22–26 h post activation, and loaded into a transfer tube and kept in a portable incubator (Minitube, Delavan, WI, USA) during transportation to the pig farm. Estrous-synchronized Yorkshire sows with natural standing estrus within 40–46 h prior to embryo transfer were used as recipients. They were anesthetized with ketamine (25 mg/kg body weight) and xylazine (1.1 mg/kg body weight) for induction and 3% of isofluorane for maintenance. Ovaries and oviducts of sows were exposed by surgery. Sows with ovaries that had freshly ruptured follicles were transferred with cloned embryos by delivering the cloned embryos into their oviducts using a syringe.

### 2.5. Observation of EGFP and RFP Expression

EGFP expression in TG pigs and their tissues was visualized by using the living organism’s fluorescent protein observation system (Model: FBL/Basic-B & N-01, BLS Ltd., Budapest, Hungary). This system consisted of a blue-light lamp with maximum excitation at 488 nm, and a red-light lamp with maximum excitation at 515 nm and goggles with light filters. Fluorescent photographs were taken under blue light by a camera equipped with light filters.

### 2.6. PCR Analysis

Genomic DNA was isolated from tail biopsies or cultured cells using a tissue or cultured cell DNA extraction kit (Omega, Doraville, GA, USA). Isolated genomic DNA was used as template to amplify the target DNA fragments by PCR (for primer sequences and amplicon length, see Supplementary Tables S1, S4 and S5). The PCR amplification products were sequenced to confirm their identities.

### 2.7. Southern Blot Analysis

**Figure 1:**
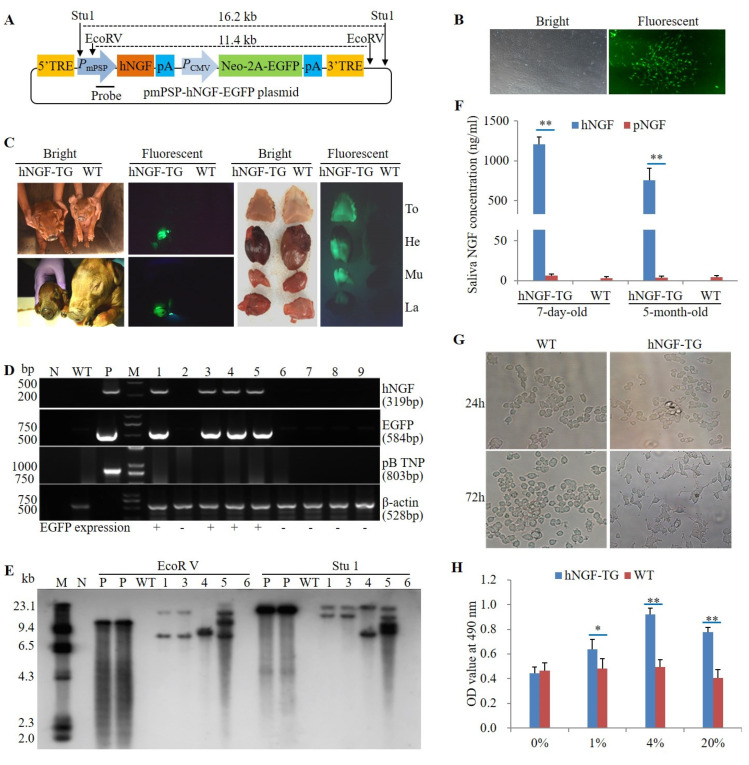
Production and identification of F_0_ generation hNGF-TG pigs. (**A**) The map of the pmPSP-hNGF-EGFP piggyBac transposon plasmid. 5′TRE and 3′TRE: the piggyBac transposon 5′ and 3′ terminal repeat elements. *P*_mPSP_: the mouse parotid secretory protein (PSP) gene promoter, which is a salivary glands-specific promoter. hNGF: the *hNGF* gene coding sequences. pA: poly A signal. *P*_CMV_: the cytomegalovirus promoter. Neo-2A-EGFP: the neomycin-resistance gene linked with the EGFP gene by a 2A peptide. The location of the probe and enzyme cutting sites for Southern blot analysis are also shown on the plasmid map. (**B**) The selected EGFP-expressing TG cell colonies that were uses as donor cells for subsequent SCNT. (**C**) EGFP expression in F_0_ hNGF-TG pigs produced by SCNT. To: tongue. He: heart. Mu: muscle. La: larynx. (**D**) PCR identification of F_0_ hNGF-TG pigs. N: negative control. WT: wild-type piglets. P: positive control using pmPSP-hNGF-EGFP or pmPB plasmid DNA as PCR template. M: markers. The ID no. of nine piglets produced by SCNT are 1, 2, 3, 4, 5, 6, 7, 8, and 9. pB TNP: piggyBac transposase gene. (**E**) Southern blot analysis of transgene integration patterns in F_0_ hNGF-TG pigs. M: markers. N: negative control. P: positive controls using pmPSP-hNGF-EGFP plasmid as sample DNA. WT: wild-type piglet. (**F**) Average hNGF and pNGF concentration in oral saliva of F_0_ hNGF-TG pigs and WT control pigs at the age of 7 days and 5 months. (**G**) Induction of neuronal differentiation of PC12 cells by hNGF-TG pig-derived oral saliva. WT and hNGF-TG: PC12 cell culture medium containing 4% of oral saliva collected at rest time from WT and hNGF-TG pigs, respectively. 24 h and 72 h: PC12 cells cultured for 24 h and 72 h, respectively. (**H**) Promotion of proliferation of TF1 cells by hNGF-TG pig-derived oral saliva. 1%, 4%, and 20%: TF1 cell culture medium containing 1%, 4%, and 20% of hNGF-TG pig-derived oral saliva, respectively. * and ** mean the difference between two groups reached statistical significance at *p* < 0.05 and *p* < 0.01, respectively.

Each pig’s tail genomic DNA (10 mg) was digested with EcoR V or Stu I and separated in a 0.8% agarose gel by electrophoresis. The DNA was subsequently transferred from the gel to a nylon membrane (GE Healthcare Life Sciences, Shanghai, China) by the capillary transfer method. The membrane was prehybridized overnight at 42 °C and then hybridized with a digoxigenin (DIG)-labeled 800 bp probe targeting the mPSP promoter of the *hNGF* gene by using a PCR DIG probe synthesis kit (Roche Applied Science, Indianapolis, IN, USA). Hybridization was performed using the DIG-High Prime DNA Labeling and Detection Starter Kit (Roche Applied Science, Shanghai, China). After hybridization, the membrane was incubated for 30 min in a blocking solution and subsequently incubated for a further 30 min in an anti-digoxigenin-AP antibody solution. The membrane was then incubated with CSPD ready-to-use solution for 5–20 min, and a photo was captured with an EC3 imaging system (UVP, Upland, CA, USA). The locations of EcoR V or Stu I restriction sites and probe binding site on the pmPSP-hNGF-EGFP plasmid are indicated in Figure 1A.

### 2.8. Saliva Collection during Rest Time

Several cotton swabs were placed into the mouth of pigs during rest time (non-feeding time) to allow them to chew and soak the cotton swabs with oral saliva. The soaked cotton swabs were then removed from the pigs’ mouths and the saliva was squeezed out from the cotton swabs into a 2 mL tube. About 100–200 µL of saliva was collected from each pig and stored at −80 °C for later use.

### 2.9. Enzyme-Linked Immunosorbent Assay (ELISA) Analysis

hNGF and pNGF protein levels in saliva or tissues were measured by the ELISA kits (EIAab Science, Wuhan, China) for hNGF (Cat. No. E0105h) and pNGF (Cat. No. E0105p), respectively, following the instructions provided by the manufacturer.

### 2.10. PC12 Cell Differentiation Assay

Undifferentiated rat PC12 cells (Abcam, Cambridge, MA, USA) were cultured for 4 days in Roswell Park Memorial Institute (RPMI) 1640 medium containing 1% fetal bovine serum (FBS) and 1% horse serum. Cells were washed and resuspended in the wells (1 × 10^4^ cells/well) of collagen-coated 6-well plates. Cells were cultured with medium containing TG pig-derived saliva or purified hNGF. The morphology of treated PC12 cells was examined under an inverted microscope and photographed.

### 2.11. TF1 Cell Proliferation Assay

Human TF1 cells (Sigma-Alrich, St. Louis, MO, USA) were cultured for 1 week in RPMI-1640 medium containing 10% FBS with 2 ng/mL rhGM-CSF (R&D System, Minneapolis, MN, USA). Cells were then washed and resuspended in RPMI-1640 + 10% FBS to a concentration of 420,000 cells/mL and replated on 96-well microplates (12,600 cells per well in 30 mL). The medium was removed 1 h after TF1 cells replating, and to the wells was respectively added 100 μL of RPMI-1640 containing TG pig-derived saliva or purified hNGF. After incubation for 40 h at 37 °C and 5% CO_2_, the medium was changed with 50 μL/well RPMI-1640 containing 10% FBS. To each well was then added 20 μL of “Cell Titer 96 Aqueous One Solution Cell Proliferation” reagent (Promega, Madison, WI, USA). The plate was incubated at 37 °C for 3 h in a humidified, 5% CO_2_ atmosphere. The absorbance of each well at 490 nm was recorded using a microplate reader (model: Synergy-H1, Bio Tek, Shoreline, WA, USA) after 3 h of incubation.

### 2.12. Inverse PCR Analysis

One microgram of genomic DNA extracted from each TG pig sample was digested with EcoR V or BamH I. The digested DNA was purified by a DNA purification column (Qiagen, Hiden, Germany) and ligated with T4 ligase overnight at 16 °C. Ligated DNA was purified via a Qiagen DNA purification column and used as a template for the PCR reaction (for primer sequences and amplicon length, see Supplementary Table S2). The resulting PCR products were ligated into a TA vector (Life Technologies, Carlsbad, CA, USA) and sequenced. The sequencing results were analyzed to obtain the genomic sequences flanking the inserted *piggyBac* transposon, which were used to blast against the sus scrofa (pig) genomic database on the NCBI BLAST website to determine the integration sites of the inserted piggyBac transposon.

### 2.13. Collection of Saliva by the Surgical and Nonsurgical Methods

**Figure 2:**
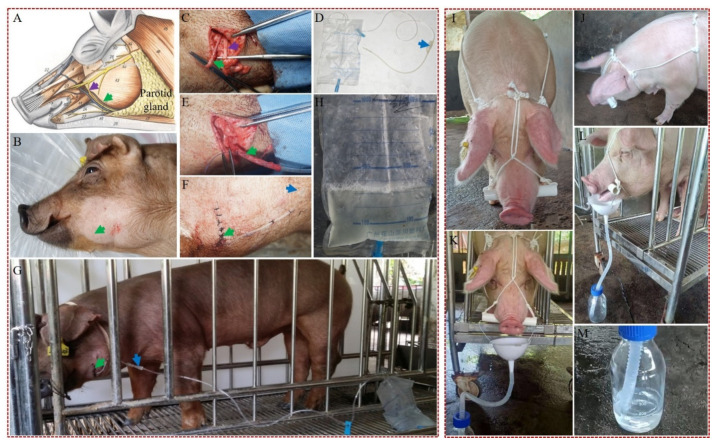
Development of a unilateral parotid duct cannulation-based (UPDCB) surgical method (left panel) and a bridle-like device-based (BLDB) nonsurgical method (right panel) for efficient collection of saliva from hNGF-TG pigs. (**A**) An anatomical drawing of the pig face showing the position of parotid gland and its duct extending to the oral cavity. (**B**) A pig that is ready (under anesthesia and with its facial hair shaved) for unilateral parotid duct cannulation surgery. (**C**) Exposure of the parotid duct after facial surgery. (**D**) A medical urethral catheter and a medical drainage bag used for the parotid duct cannulation. (**E**) Cannulation of the parotid duct with a urethral catheter. (**F**) Suture of the facial wound after the parotid duct cannulation surgery. (**G**) Collection of parotid saliva from a pig by the UPDCB method. (**H**) The TG pigs’ parotid saliva collected into a drainage bag by the UPDCB method. Green, purple, and blue arrows point to the position of parotid duct, facial nerve, and urethral catheter connector, respectively. The front (**I**) and lateral (**J**) view of a TG pig wearing a bridle-like device consisting of a headstall and a bit, which were made of a rope and a plastic pipe (about 3 cm in diameter), respectively. The bit is attached by the headstall, which keeps the bit in place in the mouth to make the mouths of TG pigs slightly open. The front (**K**) and lateral (**L**) view of a TG pig wearing a bridle-like device attaching with a simple saliva collection device, which is composed of a funnel and a bottle. (**M**) The TG pigs’ oral saliva collected into a bottle by the BLDB method. Collection of TG pigs’ oral saliva by the BLDB nonsurgical method also was shown in Supplementary Video S1.

The procedures for the collection of saliva from TG pigs by the surgical and non-surgical methods is shown in Figure 2 and Supplementary Video S1.

### 2.14. Purification of hNGF

Saliva collected from hNGF-TG pigs during their feeding time by the nonsurgical method described above was mixed and centrifuged at 11,500 rpm/min for 20 min at 16 °C. The saliva supernatant was collected, and its pH value was adjusted to 4.0 with HCl and its conductivity adjusted to 3.94 mS/cm using solution A containing 0.05 M HAC (pH = 4.0). The saliva supernatant was then filtered using a sterile 0.45µm membrane and then loaded onto a 5 mL Focurose 6FF column (Cat #: y17070701, Huiyan Bio, Wuhan, China), which was previously equilibrated with 10 CV of solution A. An aliquot of 120 mL saliva supernatant was loaded at a flow rate of 2 ml/min onto each column. After washing with 5 CV of solution A, the retained proteins in the column were eluted seriatim with solution B containing 0.05 M HAC and 0.4 M NaCl (pH = 4.0), solution C containing 0.05 M HAC and 1.0 M NaCl (pH = 4.0) and solution D containing 0.05 M Tris-HCl and 0.4 M NaCl (pH = 9.0). Each eluted protein fraction (5 mL to 10 mL) was monitored by the AKAT pure 25 chromatography system (GE Healthcare, Little Chalfont, UK) at 280 nm UV absorptance, and was collected for subsequent SDS-PAGE and Western blot analysis.

### 2.15. SDS-PAGE and Western Blot Analysis

A 30 µL aliquot of each eluted protein sample was mixed with loading buffer in the presence of 1.5% β-mercaptoethanol and loaded into each well of an 18% SDS-PAGE gel and fractionated by electrophoresis at 80 V for 90 min. Proteins in the gels were visualized by Coomassie blue staining. For Western blot analysis, proteins in the gels were transferred to a PVDF membrane at 110 V for 90 min by the Trans-Blot SD Semi-Dry Electrophoretic Transfer Cell instrument (Bio-Rad, Hercules, CA, USA). Detection of hNGF on the membrane was carried out by using polyclonal goat anti-hNGF primary antibody (Cat. #: AF-256-NA, R & D systems, Minneapolis, MN, USA), HRP-conjugated rabbit anti-goat secondary antibody (Cat. #: HAF017, R & D systems, Minneapolis, MN, USA), and SuperSignal West Pico Chemiluminescent Substrates (Thermo Scientific Pierce, Guangzhou, China) following the manufacturer’s protocols. Commercial hNGF mature peptide (Cat. #: 256-GF-100/CF, R & D systems, Minneapolis, MN, USA) was used as a positive control.

### 2.16. Liquid Chromatography-Mass Spectrometry/Mass Spectrometry (LC-MS/MS) Analysis

Purified hNGF was analyzed by electrophoresis in a polyacrylamide gel. After staining with Coomassie blue solution, the 13.5 kD hNGF protein band was removed from the gel and cut into small cubes, de-stained by ammonium bicarbonate and acetonitrile, and digested with trypsin for 10 h. The digested products were then analyzed by the LC-MS/MS system (Q Exactive Orbitrap model, Thermo Fisher Scientific, Waltham, MA, USA) for verifying the presence of hNGF.

### 2.17. Off-Target Analysis

The recognition sequences of pNGF-sgRNA 5 and pNGF-sgRNA 10 were submitted to the online CRISPOR program (http://www.crispor.tefor.net (accessed on 2 October 2020)) to predict the possible off-target sites in the selected pig genome (Sscrofa 11.1). Five potential off-target sites with a high predicted off-target score were selected and their PCR amplification primers were designed by the same CRISPOR program (see Supplementary Table S6). The PCR products of each tested potential off-target site were sequenced to verify their identity.

### 2.18. Statistical Analysis

The NGF concentration and the OD value between two groups were analyzed by one-way ANOVA using the SPSS 19.0 software (IBM Corp., Armonk, NY, USA).

## 3. Results

### 3.1. Production and Identification of F_0_ Generation hNGF-TG Pigs

A *piggyBac* transposon plasmid pmPSP-hNGF-EGFP, carrying a mouse parotid secretory protein (mPSP) gene promoter-driven *hNGF* transgene and a cytomegalovirus (CMV) promoter-controlled Neo-2A-EGFP selectable marker gene (Figure 1A), was previously constructed and tested in TG mice [31]. This plasmid was co-transfected with the *piggyBac* transposase expression plasmid pmPB [32] into a Duroc boar-derived ear fibroblast. After selection by neomycin, EGFP-expressing cell colonies were obtained (Figure 1B). Five EGFP-positive cell colonies were then randomly selected, mixed, and used as donor cells to produce nine cloned pigs by somatic cell nuclear transfer (SCNT) (Table 1). Four of the pigs generated in this manner (no. 1, no. 3, no. 4, and no. 5) were identified as the F_0_ generation hNGF-TG pigs as they were not only EGFP positive (Figure 1C), but also carried the hNGF and selectable marker transgene in their genome (Figure 1D). As indicated by the Southern blot analysis, the four F_0_ hNGF-TG pigs exhibited three different genomic integration patterns for the *hNGF* transgene cassette (Figure 1E), suggesting that four F_0_ hNGF-TG pigs were derived from three different donor cell colonies. Two of the F_0_ hNGF-TG pigs (no. 1 and no. 4) died for unknown reasons within one week after birth, which is a common phenomenon of newborn cloned animals. The other two F_0_ hNGF-TG pigs (no. 3 and no. 5) survived into adulthood with a normal phenotype.

In oral saliva collected at rest time of 7-day-old and 5-month-old hNGF-TG pigs, hNGF protein level reached 1207.41 ± 95.33 ng/mL and 757.30 ± 126.21 ng/mL, respectively, much higher than endogenous salivary pNGF protein levels at the same age (6.24 ± 2.16 ng/mL and 4.02 ± 1.45 ng/mL, respectively) (Figure 1F). hNGF-TG pig-derived oral saliva not only induced neuronal differentiation of PC12 cells but also enhanced proliferation of TF1 cells when supplemented to the culture medium of these two NGF-responsive cell lines (Figure 1G,H). These results suggest that the saliva of our hNGF-TG pigs has NGF-like bioactivities.

### 3.2. Production and Analysis of F_1_ Generation hNGF-TG Pigs

**Figure 3:**
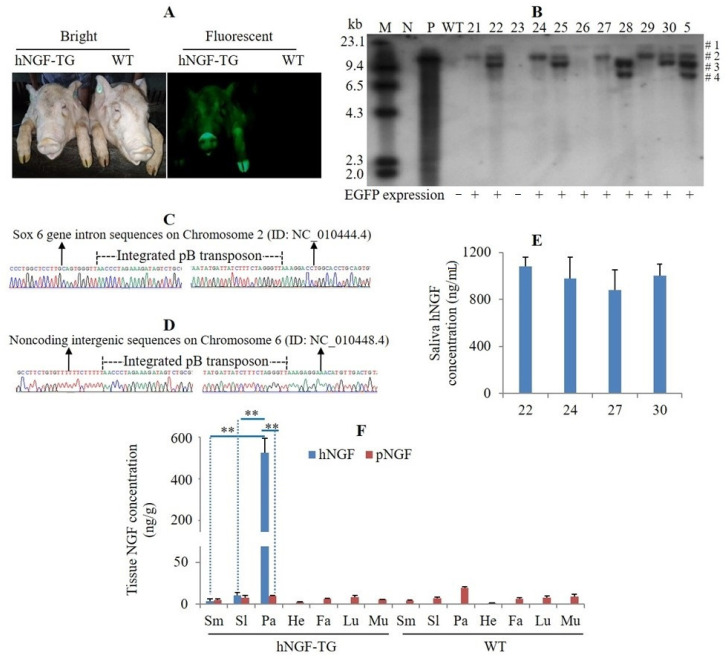
Production and analysis of F_1_ generation hNGF-TG pigs. (**A**) EGFP expression in F_1_ hNGF-TG pigs produced by mating the no. 5 F_0_ hNGF-TG Duroc boar with WT Yorkshire sows (with dominant white coat color). (**B**) Southern blot analysis of transgene integration patterns in the genome of F_1_ hNGF-TG pigs following EcoR V enzyme digestion. M: markers. N: negative control. P: positive controls using pmPSP-hNGF-EGFP plasmid as sample DNA. WT: wild-type piglets. 21, 22, 23, 24, 25, 26, 27, 28, 29, and 30 are the ID no. of 10 offspring delivered by a WT sow mated with the no. 5 F_0_ hNGF-TG boar. #1, #2, #3, and #4 are the four copies of transgene carried by no. 5 F_0_ hNGF-TG boar. (**C**,**D**) The transgene integration site identified by inverse PCR in F_1_ hNGF-TG pig no. 26 carrying the #1 copy of transgene, and no. 24 and no. 21 carrying the #2 copy of transgene, respectively. (**E**) hNGF concentration in oral saliva collected at rest time of no. 22, no. 24, no. 27, and no. 30 F_1_ hNGF-TG pigs at the age of 5 months. (**F**) Average hNGF and pNGF protein concentrations in different tissues of WT control pigs and F_1_ hNGF-TG pigs no. 22, no. 24, and no. 27 sacrificed at the age of 5 months. Sm: submandibular gland. Sl: sublingual gland. Pa: parotid gland. He: heart. Fa: fat. Lu: lung. Mu: muscle. ** means the difference between two groups reached statistical significance at *p* < 0.01.

To enlarge the hNGF-TG pig population, F_1_ hNGF-TG pigs were produced by mating the no. 5 F_0_ hNGF-TG pig (a Duroc boar) with WT Yorkshire sows. We observed EGFP expression in all the F_1_ hNGF-TG pigs (Figure 3A). A Southern blot analysis demonstrated that the putative four transgene copies of the no. 5 F_0_ hNGF-TG boar were transmitted to its TG progeny according to Mendel’s law of segregation and independent assortment, as each TG progeny randomly inherited one to three copies of the transgene from its father (Figure 3B). Through inverse PCR analysis, the integration sites of the #1 copy of transgene were identified in no. 26, and the #2 copy of transgene was identified in no. 21 and no. 24 F_1_ hNGF-TG pigs, which were carrying only one copy of the transgene (Figure 3B–D). The hNGF protein level in oral saliva collected at rest time of 5-month-old F_1_ hNGF-TG pigs was similar to that of the same age F_0_ hNGF-TG pigs (Figure 1F and Figure 2E). In F_1_ hNGF-TG pigs, hNGF protein was specifically expressed in three salivary glands, but the hNGF protein level in parotid glands (510.82 ± 83.91 ng/g) was significantly higher than that in submandibular glands (3.78 ± 2.42 ng/g) and sublingual glands (10.37 ± 3.84 ng/g) (Figure 3F). In submandibular glands and sublingual glands of hNGF-TG pigs, the internal pNGF protein level was comparable to the foreign hNGF protein level (Figure 3F). Nevertheless, in the parotid glands of hNGF-TG pigs, the internal pNGF protein abundance was much lower than the heterologous hNGF protein abundance (9.59 ± 0.92 ng/g vs. 510.82 ± 83.91 ng/g) (Figure 3F).

### 3.3. Development of Efficient Methods for Collection of Saliva from hNGF-TG Pigs

Two different techniques were developed for efficient collection of saliva from hNGF-TG pigs. The first was a surgical technique designated as “unilateral parotid duct cannulation-based (UPDCB)” (Figure 2A–G). The second technique was nonsurgical and is referred to as “bridle-like device-based (BLDB)” (Figure 2I–M and Supplementary Video S1). These two techniques were tested for harvesting saliva samples from adult hNGF-TG pigs to examine their collection efficiency.

Using the UPDCB method, an average of 512 ± 84 mL of parotid saliva with a hNGF protein concentration averaging 14.2 ± 3.9 µg/mL was collected from an adult hNGF-TG pig per day (Table 2). About 80 to 90% of the parotid saliva collected by the UPDCB method was secreted by the TG pigs during their feeding time (Table 2). The level of endogenous pNGF protein in parotid saliva collected by this UPDCB method was 0.04 ± 0.02 µg/mL, which is very low compared with that of hNGF (Table 2).

In hNGF-TG pigs, hNGF protein was mainly secreted with parotid saliva during the feeding time because it was mainly expressed in parotid glands (Figure 3F), and parotid saliva was mostly secreted during feeding time (Table 2). Therefore, the BLDB method was tested during the feeding time of hNGF-TG pigs. Using BLDB, 303 ± 76 mL of oral saliva with an average hNGF protein level of 4.5 ± 0.7 µg/mL was collected from an adult hNGF-TG pig per day within 2 h during the feeding time (Table 2). The level of endogenous pNGF protein in parotid saliva collected by the BLDB method was 0.02 ± 0.01µg/mL, which was very low compared with that of hNGF (Table 2).

### 3.4. Purification of hNGF Protein from the Saliva of hNGF-TG Pigs and Bioassay of Purified hNGF

**Figure 4:**
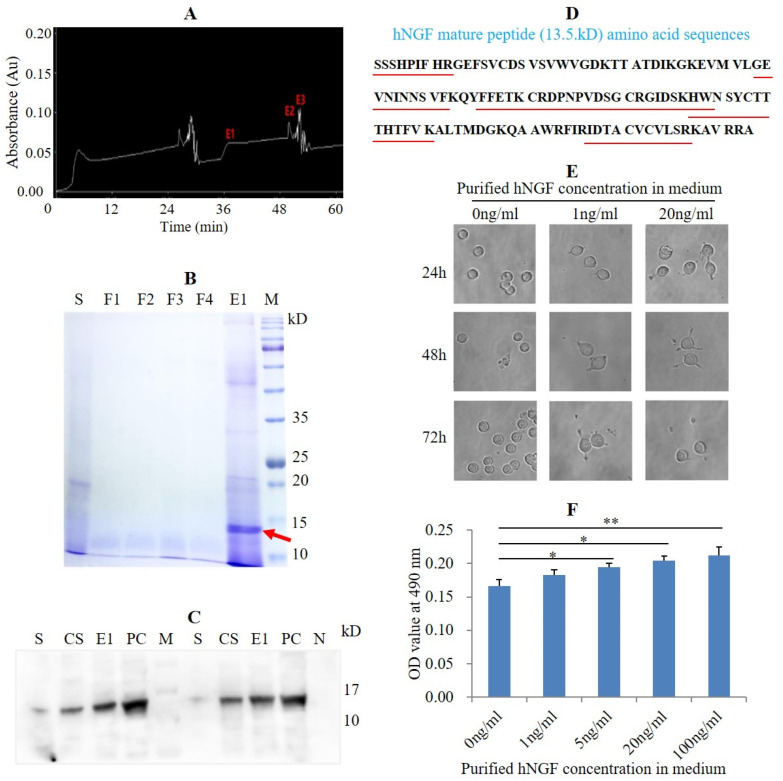
Purification of hNGF protein from hNGF-TG pigs-derived oral saliva and bioassay of purified hNGF. (**A**) Chromatogram of the salivary protein fractions eluted from the purification column. E1, E2, and E3 are three eluted protein fractions detected by UV absorbance. (**B**) SDS-PAGE analysis of eluted protein fractions. S: hNGF-TG pig-derived oral saliva. F1, F2, F3, and F4 are four fractions flow-through the column. Red arrow points to the putative hNGF mature peptide (13.5 kD) contained in the E1 fraction. (**C**) Identification of purified hNGF in the E1 fraction by Western blot. S: hNGF-TG pig-derived oral saliva. CS: concentrated hNGF-TG pig-derived oral saliva. E1: the eluted E1 protein fraction. PC: positive control (purchased commercial hNGF). M: markers. N: negative control. (**D**) Identification of the amino acid sequences of purified hNGF in the E1 fraction by mass spectrometry analysis. The amino acid sequences of trypsin digestion-generated five hNGF short peptides (with red underline), which were identified by mass spectrometry (see the mass spectrograms in Figure 5), fully match their expected amino acid sequences in the hNGF mature peptide. (**E**) Induction of neuronal differentiation of PC12 cells by purified hNGF. (**F**) Promotion of proliferation of TF1 cells by purified hNGF. * and ** mean the difference between two groups reached statistical significance at *p* < 0.05 and *p* < 0.01, respectively.

**Figure 5:**
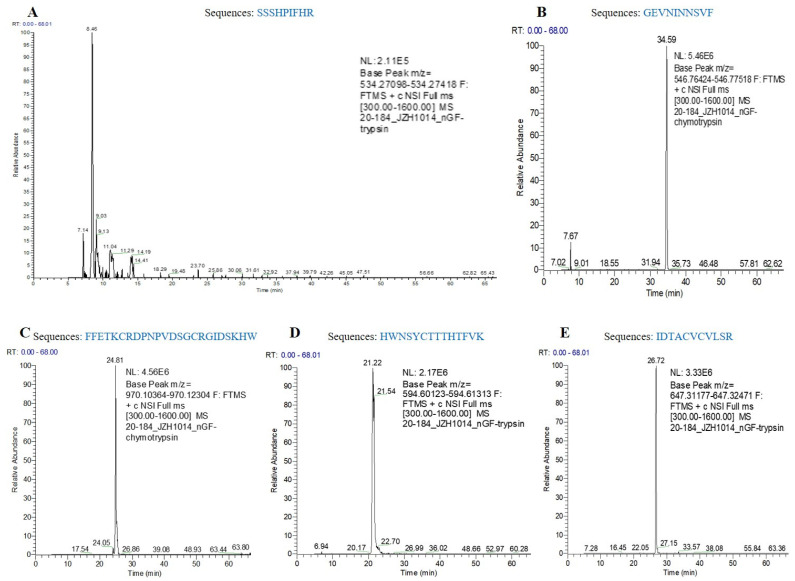
The mass spectrograms of amino acid sequence analysis of purified hNGF. (**A**–**E**) are mass spectrograms of analysis of five different short peptides generated by trypsin digestion of purified hNGF.

hNGF protein was purified from the hNGF-TG pig-derived oral saliva by the size-exclusion chromatography method (Figure 4A). SDS-PAGE analysis demonstrated that the eluted protein fraction E1, obtained after the purification, contained a putative 13.5 kD protein (Figure 4B), which matches the molecular weight of the hNGF mature peptide. A Western blot analysis showed that the 13.5 kD hNGF mature peptide was contained in the eluted protein fraction E1 (Figure 4C), which was then confirmed by mass spectrometry-based amino acid sequence analysis (Figure 4D and Figure 5). The purification efficiency of salivary hNGF protein was 43.3% (the total amount of purified hNGF protein/the total amount of hNGF protein in collected oral saliva). The purity of hNGF protein isolated from oral saliva was 62.1% (the amount of hNGF protein in the eluted protein fraction E1/the amount of total proteins in the eluted protein fraction E1).

Addition of hNGF purified from the saliva of hNGF-TG pigs to the cell culture medium not only induced neuronal differentiation of PC12 cells but also promoted proliferation of TF1 cells (Figure 4E,F). These results indicate that the purified hNGF was functional in NGF-responsive cells.

### 3.5. Production of Double-Transgenic hNGF-TG/ pNGF-KO Pigs

The ratio of endogenous pNGF protein to hNGF protein in oral saliva collected at rest time, and in parotid saliva and oral saliva collected during feeding time in our hNGF-TG pigs was lower than 1% (Figure 1F and Table 2). Even at this low ratio, it is possible that endogenous pNGF will be purified together with hNGF as both proteins have a high homology. If the purified hNGF is “contaminated” by endogenous pNGF, it may induce immunogenic responses when administered to patients. This problem can be avoided by knocking out the endogenous *pNGF* gene of hNGF-TG pigs. However, knockout of the endogenous *pNGF* gene may cause negative effects on hNGF-TG pigs because the previously integrated *hNGF* transgene may be unable to compensate for the functions of the endogenous *pNGF* gene, as they are driven by different promoters. Therefore, we chose to replace the *pNGF* gene with an *hNGF* gene, leaving its promoter intact, by the CRISPR/Cas9 system combined with homologous recombination.

**Figure 6:**
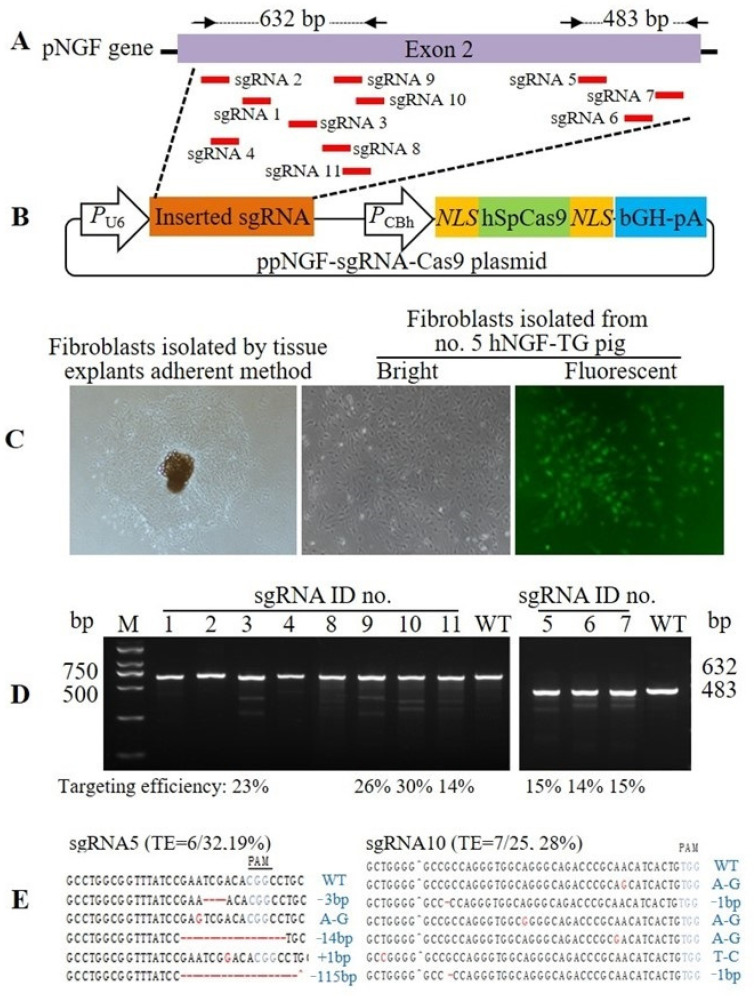
Design and selection of sgRNAs for efficient targeting of the *pNGF* gene via the CRISPR/Cas9 system. (**A**) Location of the recognition sites of 11 designed sgRNAs that target the *pNGF* gene. (**B**) The map of the CRISPR/Cas9 system expression plasmid (ppNGF-sgRNA-Cas9) that targets the *pNGF* gene. (**C**) Ear fibroblasts isolated from no. 5 F_0_ hNGF-TG pig for the second round of genetic modification. (**D**) T7E1 assay of the targeting efficiency of 11 designed sgRNAs. 1–11: the ID no. of 11 designed pNGF-targeting sgRNAs. (**E**) Analysis of the targeting efficiency of sgRNA5 and sgRNA10 by sequencing the PCR amplification products of their targeting sites following transfection of the ppNGF-sgRNA5-Cas9 and ppNGF-sgRNA10-Cas9 plasmids into pig fibroblasts. TE, targeting efficiency.

Eleven sgRNAs that target the *pNGF* gene were designed, synthesized, and respectively cloned into a pX330-U6-Chimeric-BB-CBh-hSpCas9 plasmid to construct 11 ppNGF-sgRNA-Cas9 plasmids (Figure 6A,B). These 11 pNGF-targeting CRISPR/Cas9 system expression plasmids were then transfected into no. 5 F_0_ hNGF-TG pig-derived ear fibroblasts (Figure 6C) to examine their targeting efficiency. A T7 endonuclease I (T7EI) mismatch detection assay showed that the targeting efficiency ranged from 0 to 30% (Figure 6D). The targeting efficiency of sgRNA5 and sgRNA10 was confirmed by analyzing the mutation rate on their targeting sites following transfection of the ppNGF-sgRNA5-Cas9 and ppNGF-sgRNA10-Cas9 plasmids into pig fibroblasts (Figure 6E). Finally, sgRNA5 and sgRNA10 were chosen for subsequent targeting of the *pNGF* gene in no. 5 F_0_ hNGF-TG pig.

**Figure 7:**
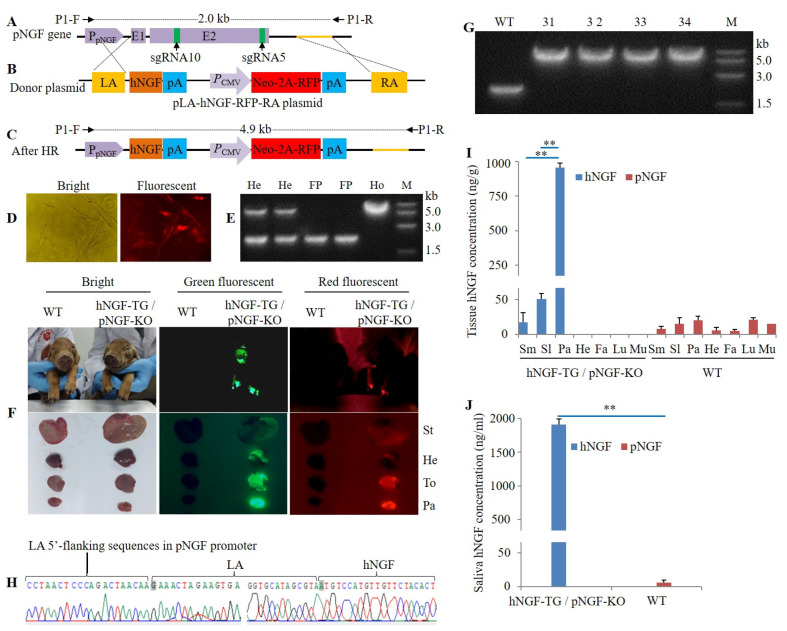
Production of double-transgenic hNGF-TG/pNGF-KO pigs. (**A**) The endogenous *pNGF* gene locus. (**B**) The map of the donor plasmid pLA-hNGF-RFP-RA used for replacement of the endogenous *pNGF* gene with the foreign *hNGF* gene by CRISPR/Cas9 system combined with homologous recombination (HR). LA and RA: left and right homologous arms, respectively. (**C**) The endogenous *pNGF* gene locus after HR. P1-F and P1-R are a set of primers used for PCR identification of clones with its endogenous *pNGF* gene being replaced by the foreign *hNGF* gene. (**D**) RFP-expressing pig fibroblast cell colonies resulted from selection of the no. 5 F_0_ hNGF-TG pig-derived fibroblasts co-transfected with the donor plasmid pLA-hNGF-RFP-RA, and the CRISPR/Cas9 system expression plasmids (ppNGF-sgRNA5-Cas9 and ppNGF-sgRNA10-Cas9) that target the *pNGF* gene. (**E**) PCR identification of selected RFP-expressing fibroblast cell colonies with their endogenous *pNGF* gene being replaced by the foreign *hNGF* gene. He: heterozygous cell colonies. FP: false positive. Ho: homozygous cell colonies, which were used as donor cells for subsequent SCNT. (**F**) EGFP and RFP expression in double-transgenic hNGF-TG/pNGF-KO pigs generated by SCNT. St: stomach. He: heart. To: tongue. Pa: parotid gland. (**G**) PCR identification of hNGF-TG/pNGF-KO pigs with their endogenous *pNGF* gene being replaced by the foreign *hNGF* gene. WT: wild-type pig. 31, 32, 33, and 34 are the ID no. of four hNGF-TG/pNGF-KO pigs generated by SCNT. (**H**) The sequencing result of the PCR amplification product of the endogenous *pNGF* gene locus in hNGF-TG/pNGF-KO pigs. (**I**) Average hNGF and pNGF protein concentration in different tissues of hNGF-TG/pNGF-KO pigs (no. 31 and no. 32) died within one day after birth. No pNGF was detected in examined tissues of hNGF-TG/pNGF-KO pigs. Sm: submandibular gland. Sl: sublingual gland. Pa: parotid gland. He: heart. Fa: fat. Lu: lung. Mu: muscle. (**J**) Average hNGF and pNGF concentration in oral saliva collected at rest time from survived hNGF-TG/pNGF-KO pigs (no. 33 and no. 34) at the age of 5 days. No pNGF was detected in oral saliva of hNGF-TG/pNGF-KO pigs. ** means the difference between two groups reached statistical significance at *p* < 0.01.

**Figure 8:**
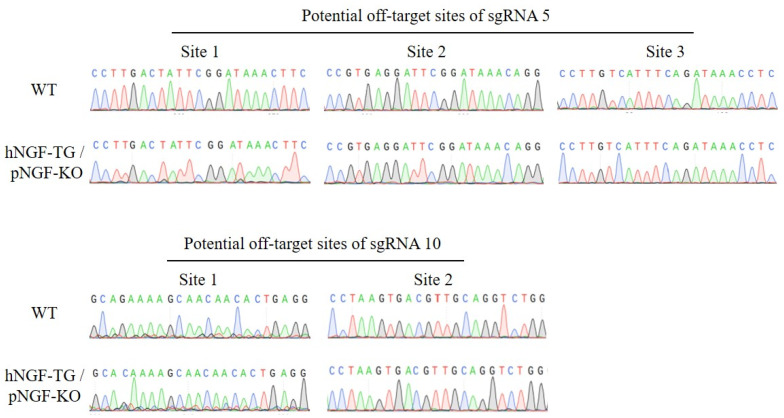
Off-target analysis of double-transgenic hNGF-TG/pNGF-KO fibroblast cell colonies.

A homologous recombination donor plasmid called pLA-hNGF-RFP-RA, which contains a left arm and a right arm homologous to the internal *pNGF* gene, was constructed (Figure 7A,B). CRISPR/Cas9-mediated homologous recombination was then used to substitute the endogenous *pNGF* gene with the *hNGF* gene (Figure 7C). Specifically, following neomycin-based selection of fibroblasts co-transfected with pLA-hNGF-RFP-RA, ppNGF-sgRNA5-Cas9, and ppNGF-sgRNA10-Cas9 plasmids, RFP-expressing cell colonies were isolated (Figure 7D). Homozygous cell colonies with double alleles of endogenous *pNGF* gene successfully substituted by the foreign *hNGF* gene were identified by PCR (Figure 7E). An off-target assay indicated that in one homozygous cell colony, sgRNA5, and sgRNA10-mediated targeting of the *pNGF* gene did not cause mutation in any of the five predicted potential off-target sites (Figure 8). This homozygous cell colony was then used as donor cells to produce four double-transgenic hNGF-TG/pNGF-KO pigs by SCNT (Table 3). Two of the hNGF-TG/pNGF-KO pigs died within one day after birth, while the other two survived with a normal phenotype. These double-transgenic pigs expressed both marker genes, EGFP and RFP (Figure 7F). PCR analysis and sequencing confirmed that the endogenous *pNGF* gene in all four hNGF-TG/pNGF-KO pigs was replaced by the foreign *hNGF* gene (Figure 7G,H).

In our double-transgenic hNGF-TG/pNGF-KO pigs, hNGF protein was not only selectively synthesized in the three salivary glands, at a much higher level in parotid glands compared to submandibular or sublingual glands (Figure 7I), but also secreted at high levels into the saliva (Figure 7J). In contrast, no pNGF protein was detected in the tissues and saliva of these double-transgenic pigs (Figure 7I,J), suggesting that the *pNGF* gene was successfully replaced by the *hNGF* gene, and these double-transgenic pigs can be used as efficient bioreactors to produce pharmaceutically valuable hNGF protein.

## 4. Discussion

Here we describe the successful production of a valuable therapeutic protein, hNGF, by using the salivary glands of TG pigs as efficient bioreactors. The concentration of the hNGF protein in oral saliva collected at rest time in our hNGF-TG pigs was similar to the concentration of hNGF in the saliva of a hNGF-TG mouse line, which we previously generated using the same *PSP-hNGF* transgene construct [31]. This finding demonstrates that the same *PSP-hNGF* transgene expression cassette gave rise to similar hNGF expression levels in two different species. However, as the daily volume of saliva secreted by an adult pig is approximately 10,000 times higher than that secreted by an adult mouse [10,35], salivary glands of TG pigs are far more efficient expression systems for the production of hNGF. Similarly, while the hNGF production efficiency in oral saliva (4.5 µg/mL) and in parotid saliva (14.2 µg/mL) of hNGF-TG pigs was lower than that in the milk of TG rabbits (50 to 250 µg/mL) [36], salivary glands of TG pigs are still superior bioreactors as the saliva secreting period of pigs is much longer than the lactating period of rabbits, and the daily volume of saliva secreted by the pigs is also much higher than the daily volume of milk lactated by the rabbits (15 L/day/pig vs. 0.17–0.22 L/day/rabbit) [4,10].

At present, the cost of the approved bacteria-produced hNGF protein for treating neurotrophic keratitis is high. It is about 12.9 GBP/µg, since a typical eight-week treatment of neurotrophic keratitis with the Cenegermin (OXERVATE^®^) drug containing 1.12 mg of bacteria-synthesized hNGF costs GBP 14,500 [22]. Considering that the prevalence of neurotrophic keratitis was estimated as 1.6 per 10,000 people [37], the total number of neurotrophic keratitis patients in the country with the largest population, China, is predicted to be about 220,000, which requires approximately a total of 250 g of hNGF protein for treatment. Using the nonsurgical method, we could collect 303 mL of oral saliva containing 4.5 µg/mL of hNGF protein from an adult hNGF-TG pig per day. That means an adult hNGF-TG pig can synthesize about 500 mg of hNGF per year; even if only a half of the expressed hNGF protein can be purified from the saliva of hNGF-TG pigs, then just 500 adult hNGF-TG pigs can provide enough hNGF protein within two years for treating all the estimated 220,000 neurotrophic keratitis patients in China.

The cloning efficiency in this study is about 0.3% (number of born cloned pigs/number of transferred 2-cell stage cloned embryos), which is quite low. However, the current overall pig cloning efficiency is also very low in the world because no reliable method that can dramatically enhance the full-term developmental rate of cloned pig embryos has been reported so far. The average full-term developmental rate of a very large number (about 100,000) of cloned pig embryos generated in our laboratory during 2009 to 2011 was similar with that in the present study [38]. The developmental efficiency of cloned pig embryos could be affected by many factors, especially the quality or status of donor cells. In this study, the developmental competence of a cloned pig embryo might be negatively affected by the genetic modification and the long-term drug treatment of donor cells, which have been found to cause adverse effects on pig cloning efficiency [39,40]. Although the cloning efficiency is low in this study, we still obtained a small number of live transgenic founder pigs, which can be used to produce a large number of transgenic offspring for further investigation by sexual reproduction.

Although in the first round of genetic engineering, the *hNGF* transgene was randomly integrated into the genome of host TG pigs by *piggyBac* transposon-mediated gene transfer, it was inserted at the noncoding genomic regions of some host TG pigs, as identified by inverse PCR. The hNGF protein was selectively synthesized in the salivary glands, although with a much higher level in parotid glands than in submandibular and sublingual glands of hNGF-TG pigs. This indicated that the promoter of mouse parotid secretory protein gene used in the present study is a suitable regulatory element for driving specific transgene expression in mammalian salivary gland bioreactors. The surviving hNGF-TG pigs showed a normal phenotype and normal fertility, suggesting that the *piggyBac* transposon-mediated integration of *hNGF* transgene and the heterologous expression of hNGF protein in salivary glands have no negative side effect on hNGF-TG pigs. The survival and normal development of the double-transgenic hNGF-TG/pNGF-KO pigs also suggested that replacing the endogenous *pNGF* gene with an additional copy of *hNGF* transgene in the second round of genetic modification did not cause negative effect.

The hNGF synthesis rate in the saliva of hNGF-TG pigs can be further improved by mating the heterozygous hNGF-TG pigs to each other. The produced homozygous offspring would then contain more copies of the *hNGF* transgene, presumably increasing hNGF synthesis. In addition, administration of epinephrine and norepinephrine to hNGF-TG pigs may also increase the hNGF production efficiency as these adrenergic agonists can dramatically increase salivary NGF protein concentration in animals by promoting NGF protein secretion into saliva [41,42].

The saliva collection efficiency of the nonsurgical BLDB and the surgical UPDCB methods used in this study was comparable. However, we observed that all of the three tested hNGF-TG pigs lost the catheter connected to their parotid ducts 5 to 20 days post-surgery by rubbing their cheeks on the cage or wall, suggesting that the surgical approach needs to be improved. Furthermore, the invasive surgical method may raise concerns about animal welfare. In contrast, using the nonsurgical BLDB saliva collection procedure, no negative effect on the digestion or physiology of the hNGF-TG pigs was observed during a one-month testing period. This finding might be due to the fact that the volume of the oral saliva collected by the BLDB method (303 mL/day/pig) only accounts for 2% of the daily volume of saliva secreted by an adult pig (15 L/day/pig) [10], and hence does not cause any disturbance to treated pigs. Therefore, the BLDB method is a promising nonsurgical approach for the collection of a large volume of saliva from TG pigs for the purification of target proteins.

Size-exclusion chromatography was used in the present study to separate hNGF protein from the saliva of hNGF-TG pigs. This purification method has the advantage of mild extraction conditions, simple operation, isocratic elution, and easy to scale-up. However, the purification efficiency of salivary hNGF protein (43.3%) and the purity of isolated hNGF protein (62.1%) resulted from the size-exclusion chromatography-mediated purification need to be improved. In future experiments, antibody-based immunoaffinity chromatography can be used to test whether it can offer improved hNGF protein isolation efficiency and purity.

We found mature hNGF protein purified from the saliva of TG pigs to be functional in inducing neuronal differentiation of PC12 cells and promoting proliferation of TF1 cells, indicating that the hNGF protein had been properly processed, modified, and secreted in hNGF-TG pigs. In addition to NGF, many other clinically important proteins, such as immunoglobulins, antimicrobial peptides, and growth factors, are naturally synthesized in the salivary glands and secreted into the saliva of mammals [43,44,45,46]. Therefore, the salivary glands of TG pigs may serve as ideal expression platforms for the synthesis of other therapeutically important proteins.

In summary, we have demonstrated efficient production of biologically active hNGF protein from the saliva of TG pigs, suggesting that salivary glands of TG livestock can serve as powerful bioreactors for the synthesis of functional pharmaceutical proteins. The study presented here develops a new and robust approach for large-scale production of high-quality therapeutic proteins, which will be beneficial for both human health and biomedicine.

## Figures and Tables

**Table 1:** Summary for production of F_0_ generation hNGF-TG pigs.

No. of Cultured (Activated)/Transferred (2-Cell Stage) Cloned Embryos	No. of Total/Farrowed Recipient Sows	No. of Transferred Cloned Embryos Per Recipient	No. of Total Born Pigs (TG/WT)	No. of Survived Pigs (TG/WT)
4650/3296	16/3	200–220	9 (4/5)	4 (2/2)

**Table 2:** Comparison of two saliva collection methods.

Collection Methods	Type of Collected Saliva	Volume of Collected Saliva (mL/day/pig)	hNGF/pNGF Concentration (µg/mL)
UPDCB (Surgical) *	Parotid saliva	512 ± 84 ^†^	14.2 ± 3.9/0.04 ± 0.02
BLDB (Nonsurgical) ^‡^	Oral saliva	303 ± 76 ^§^	4.5 ± 0.7/0.02± 0.01

The hNGF-TG pigs used for testing the saliva collection methods were five to six months old. They were fed two meals (about 1.2 kg of feed per meal) per day at 8:00 am and 4:00 pm, respectively. * The operation of the surgical saliva collection method is shown in Figure 2. Formal saliva collection testing was conducted on TG pigs at 3 days post-surgery. ^†^ 80 to 90% of the total volume of collected parotid saliva was secreted by the TG pigs during their feeding time (about 18 min per meal). ^‡^ The operation of the nonsurgical saliva collection method is shown in Figure 3 and Supplementary Video S1. ^§^ The volume of total oral saliva collected within 2 h during feeding per day (1 h per meal). For testing of the nonsurgical method, TG pigs were fed with about 0.6 kg of feed at the beginning of each meal to induce parotid saliva secretion, after their oral saliva was collected by the BLDB method for a half hour, TG pigs were again fed with about 0.6 kg of feed, and then their oral saliva was collected by the BLDB method for another half hour. Before formal saliva collection testing, TG pigs were trained for 7 days to let them get used to wearing the nonsurgical saliva collection device.

**Table 3:** Summary for production of double-transgenic hNGF-TG/pNGF-KO pigs.

No. of Cultured (Activated)/Transferred (2-Cell Stage) Cloned Embryos	No. of Total/Farrowed Recipient Sows	No. of Transferred Cloned Embryos Per Recipient	Total Number of Born Pigs	Number of Surviving Pigs
1506/1098	5/1	200–220	4	2

## Data Availability

All data are available in the main text and the supplementary materials.

## Supplementary Materials

The following supporting information can be downloaded at https://www.mdpi.com/article/10.3390/cells11152378/s1, Table S1: Sequences of primers for PCR analysis of hNGF-TG pigs; Table S2: Sequences of primers for inverse PCR analysis of transgene, Table S3: Sequences of 11 designed pNGF-targeting sgRNAs, Table S4: Sequences of primers for T7E1 assay, Table S5: Sequences of primers for PCR analysis of double-transgenic hNGF-TG/pNGF-KO cells and pigs, Table S6: Sequences of expected PCR amplicon for off-target analysis, Video S1: Collection of saliva from TG pigs by the nonsurgical method.
