# Supplementary material for: Genetically Engineered Pigs as Efficient Salivary Gland Bioreactors for Production of Therapeutically Valuable Human Nerve Growth Factor
Source: Cells. 2022 Aug 2;11(15):2378. doi: 10.3390/cells11152378 (PMC9368069; doi:10.3390/cells11152378)
Supplement: Supplementary file 1 [file cells-11-02378-s001.zip › Supplementary files/Supplementary tables.pdf]

Table S1. Sequences of primers for PCR analysis of hNGF-TG pigs

Gene	Primer sequences (5'-3')	Amplicon length (bp)
β-actin	F: CACTGCCGCATCCTCTTCCT<br>R: CTCCTGCTTGCTGATCCACATC	528
hNGF	F: GAACTCATATTGTACCACGACT<br>R: CCCCAGAATAGAATGACACC	319
EGFP	F: GCCACAAGTTCAGCGTGTCC<br>R: CATGTGATCGCGCTTCTCGTT	584
PB-transposase	F: CAGATGCCTGAGGATGGAC<br>R: CGTTGTGGCTGTAGATGATGA	803

Table S2. Sequences of primers for inverse PCR analysis of transgene integration sites in the genome of hNGF-TG pigs

Primer name	Primer sequences (5'-3')
EcoRV-LA-F	GCATGATTATCTTTTACGTGAC
EcoRV-LA-R	CTGAGATGTCCTAAATGCAC
EcoRV-RA-F	CGACCACTACCAGCAGAACACC
EcoRV-RA-R	CCACCAAGTCCTAGGCCCAT
BamHI-LA-F	GATGTAGCCCTCAAAGTCTC
BamHI-LA-R	CTTATTATGCTGTCGGGTCA
BamHI-RA-F	CCACTACCAGCAGAACACC
BamHI-RA-R	CTTCAGTGACAACGTCGAGCA
IN-EcoRV-RA-F	CCGTGCCTTCCTTGACCCTG
IN-EcoRV-RA-R	CCACCAAGTCCTAGGCCCAT

Table S3. Sequences of 11 designed pNGF-targeting sgRNAs

sgRNA name	Sequences (5'-3')
sgRNA1-F	CACCGGGACATTGCTCTCGGTGTG
sgRNA1-R	AAACCACACCGAGAGCAATGTCCC

sgRNA2-F	CACCGATCAGAGTGTAGAACAACA
sgRNA2-R	AAACTGTTGTTCTACACTCTGATC
sgRNA3-F	CACCGTTTAGTCCAGTGGGCTTGG
sgRNA3-R	AAACCCAAGCCCACTGGACTAAAC
sgRNA4-F	CACCGCTCTTCTGATCGGCGTAC
sgRNA4-R	AAACGTACGCCGATCAGAAGAGC
sgRNA5-F	CACCGCGGTTTATCCGAATCGACA
sgRNA5-R	AAACTGTCGATTCGGATAAACCGC
sgRNA6-F	CACCGCGTGCTCAGCAGGAAGGCG
sgRNA6-R	AAACCGCCTTCCTGCTGAGCACGC
sgRNA7-F	CACCGTGCGTGCTCAGCAGGAAGG
sgRNA7-R	AAACCCTTCCTGCTGAGCACGCAC
sgRNA8-F	CACCGGCTGGGGCGATAGCCGCC
sgRNA8-R	AAACGGCGGCTATCGCCCCAGCC
sgRNA9-F	CACCGGCTGGGGCGATAGCCGCCA
sgRNA9-R	AAACTGGCGGCTATCGCCCCAGCC
sgRNA10-F	CACCGCAGACCCGCAACATCACTG
sgRNA10-R	AAACCAGTGATGTTGCGGGTCTGC
sgRNA11-F	CACCGGGGCGATAGCCGCCAGGG
sgRNA11-R	AAACCCCTGGCGGCTATCGCCCC

Table S4. Sequences of primers for T7E1 assay

Primer name	Sequences (5'-3')	Amplicon length (bp)
pNGF-E2-1F	AATGGTCAATTCCCGGTCCC	632
pNGF-E2-1R	ACCTCCTTGCCCTTGATGTCC	
pNGF-E2-2F	CAAGCGGTCGTCATCCCAC	483
pNGF-E2-2R	ACTATAAATTACCATGCAGTCCT	

Table S5. Sequences of primers for PCR analysis of double-transgenic hNGF-TG/pNGF-KO cells and pigs

Primer name	Sequences (5’-3’)	Amplicon length (bp)
P1-F	CTGAGGCTTCAAGACAAGTCCC	WT: 2000,
P1-R	TTTTCAGCCAAAAGCAGACCTCC	hNGF-TG/pNGF-KO: 4900

Table S6. Sequences of expected PCR amplicon for off-target analysis

SgRNA ID no.	Potential off-target sites	Sequences of expected PCR amplicon containing the primers (with underline) and the potential off-target sites (with bold font)
sgRNA 5	mm4__1 258.00 Mbp_1_257997134	<u>GTGTAGGTTGCCAGACCCTC</u>TAATAAACTGGCCCAGAGTCGGGTGAGTCATGGTTGCACCTCCC<br>AAGTCACAGGCTATCCTCACAAGCTGTGCACCTAATTAAACTCTGATAATCTGTATAAATTAGC<br>ATTTGCTAGTGATAAATATCACGTACCAAGCCTTAAATAATACATTAGCCTTAATTGAATAAATG<br>CATATATTATGGCCTTCTAATGCATATTTGCCATAATGAGTTATCTTTTATGCACAAACAAACAG<br>CAATTCTGACTAATCAGGTGGGGTAATTGTGCTATTAAAATTCCAGGTTTCCTGAGAAAGGTCT<br>CAGTGATGGTCAGATGACAGAAAAGNNNNNNNCATCAACAACAAAACATTATTT**CCTTGACTA**<br>**TTCGGATAAACTTC**TTATTTGGGTATTTCACTACCTTTGAACCCCTAGAGTTTTGTAATTAATAT<br>CAACG<u>TCTATACCCCTTTCCCACTCCC</u>
	mm4__4 14.53 Mbp_4_14533954	<u>GGACAGGAAGACCAGCCTTC</u>CCACCTGGGCCACTGGGGAGCACCCCAGCCACTACATGGCTGCC<br>GATGTGAAGTCAGTAAGAACTTCTCTCTCGTGTTCTATGTCACACCCTCCGTGAACCAGAGGACC<br>TGAACCACCAAGGGAACAAATTCACCATTTCCACTGTAACCAAACAGAAGGAGAAATGCTTGTC<br>ATTTTCTTCCTTCTGCTCTTGCCAAGTATCCATCAGAAACCTATTTAATGTGACTATAAGGTCAGA<br>ACAAAAAGAAACTAGCAAAAGGTCTATTTGGGGAATACTACATTTTTCTTTAAATCCATAAGTTA<br>GCTTCTAACATGCATAAGTT**CCGTGAGGATTCGGATAAACAGG**AAGTCCAACTTCAAATCCCTT<br>GTCTTTAGGTCAGACCACAGATCTACTGCACAGTATGAGGTCA<u>ATCCAAGGACATCAGGCTGGA</u>
	mm4__4 22.71 Mbp_4_22706127	<u>GAGGGCCCTGGTTTAGCAAA</u>TATCTTTGTACTATTCATTTCAAGAGTAAAGGGTTTCCTTATAAAA<br>CTCAGAACATAGTTTGAAAACTTTTCTGTGATTCAGATCCTTCTCCTG**CCTTGTCATTTCAGATAA**<br>**ACCTC**TGGTTCTAAAGCACTAATGTTCCTCTAGTGTTCTATTAACTTAGATGTTTTTCTATAATTTT<br>AAACAAATCTAAGTCAAATGAAAATTTAAAAATATGGATTTCAAACAAACGGTTTAACAAGAAAT

		GTCTACTCTATTACACTTGTGACAAATCTGGTTAAGACCTCATAAGAATTATATAATTGGCTTCAG<br>AACAGAAACAATTTTTCATTAATATAACTAGCAAAAATATTTCTATAATCTCTTCTTGCCCCCATAT<br>GAAAATCAGTTAAGGGAAAGCTCAGCTGGC
sgRNA 10	mm4__14 55.82<br>Mbp_14_55822569	TAAAGTGGGAATGGTGCCCCACACCTCCTGAGAACGAGAGTCTGGTGAGTTTGCACCAAGATGAG<br>AACTGCCATTTTAAGAGCAGCTTTCCCCTCCTCAAATACCAGGCTCTCCCTTCCAGAAAGCCCCAG<br>ACTTCCCAGCTTCCTTCTGCTGAGAGATTAATGATCTGCTTCCAGGGGGCCACAGGTGCCCCGGCT<br>GTCCACTTAGATGAGCATAAAAATCCCCAAA**GCAGAAAAGCAACAACACTGAGG**GTCTCCTTTG<br>ACACCCAAGGGAAACTGCCCCAGAGGAAAGCCCTGCAGGCCAGGGCACGGGCACAAGTATACGA<br>ACTGAGGCAAAGTGACTGGTATCTAGGATGCGCTGCAAGAAATTCCTCGTTGGGTCTGTCTTTGTC<br>TGGATCCTGATGTCCACCG
	mm4__1 253.31<br>Mbp_1_253306534	TGCTTGAATGGATGCATGTGGGGCCCAGCAGGACTTGATGGATTAGCCGTAGACTGGGGAACAGG<br>TGTCAAGGATTGTACTCAGGTTTCAGGCTTGTGCACATGGATAGGTCCTGGAGGTGCTGACTGGGT<br>GGGAGAGCAGGTTTGTGGGGACAGA**CCTAAGTGACGTTGCAGGTCTGG**GTCCATGCATTGAGCA<br>GGCTCTTAGGTGTGTTAGGGTTCACAGAAGCCGTTGGAGATGTGATTCTGATGCCTTCTCATTCACC<br>ATCTGTAAAACTTCATTTTCTACTTGTTGAAGGTATGATATGTTTAGAAAAAATAATTTATATTTGCA<br>TATTTTGGATTGCCAACTTGTCTTCTTCAATCAGTTTTCTAGGCCTTTGGGATTGTACCC
